# Effect of tryptase inhibition on joint inflammation: a pharmacological and lentivirus-mediated gene transfer study

**DOI:** 10.1186/s13075-017-1326-9

**Published:** 2017-06-06

**Authors:** Alexandre Denadai-Souza, Camilla Moreira Ribeiro, Corinne Rolland, Anne Thouard, Céline Deraison, Cristoforo Scavone, Daniel Gonzalez-Dunia, Nathalie Vergnolle, Maria Christina Werneck Avellar

**Affiliations:** 1 Department of Pharmacology, Universidade Federal de São Paulo – Escola Paulista de Medicina (UNIFESP-EPM), Rua 03 de Maio, São Paulo, 04044-020 Brazil; 2 IRSD, Université de Toulouse, INSERM, INRA, ENVT, UPS, Toulouse, France; 3 INSERM, U1043, Centre de Physiopathologie de Toulouse-Purpan (CPTP); CNRS, U5282; Université Toulouse III “Paul Sabatier”, Toulouse, France; 4 Department of Pharmacology, Universidade de São Paulo, São Paulo, Brazil; 5 Department of Physiology and Pharmacology, University of Calgary, Faculty of Medicine, Calgary, AB Canada

**Keywords:** Tryptase, β-defensin, SPAG11B, APC366, Protease inhibitor, Inflammation, Synovial joint, Rheumatoid arthritis

## Abstract

**Background:**

Increasing evidences indicate that an unbalance between tryptases and their endogenous inhibitors, leading to an increased proteolytic activity, is implicated in the pathophysiology of rheumatoid arthritis. The aim of the present study was to evaluate the impact of tryptase inhibition on experimental arthritis.

**Methods:**

Analysis of gene expression and regulation in the mouse knee joint was performed by RT-qPCR and in situ hybridization. Arthritis was induced in male C57BL/6 mice with mBSA/IL-1β. Tryptase was inhibited by two approaches: a lentivirus-mediated heterologous expression of the human endogenous tryptase inhibitor, sperm-associated antigen 11B isoform C (hSPAG11B/C), or a chronic treatment with the synthetic tryptase inhibitor APC366. Several inflammatory parameters were evaluated, such as oedema formation, histopathology, production of IL-1β, -6, -17A and CXCL1/KC, myeloperoxidase and tryptase-like activities.

**Results:**

*Spag11c* was constitutively expressed in chondrocytes and cells from the synovial membrane in mice, but its expression did not change 7 days after the induction of arthritis, while tryptase expression and activity were upregulated. The intra-articular transduction of animals with the lentivirus phSPAG11B/C or the treatment with APC366 inhibited the increase of tryptase-like activity, the late phase of oedema formation, the production of IL-6 and CXCL1/KC. In contrast, neutrophil infiltration, degeneration of hyaline cartilage and erosion of subchondral bone were not affected.

**Conclusions:**

Tryptase inhibition was effective in inhibiting some inflammatory parameters associated to mBSA/IL-1β-induced arthritis, notably late phase oedema formation and IL-6 production, but not neutrophil infiltration and joint degeneration. These results suggest that the therapeutic application of tryptase inhibitors to rheumatoid arthritis would be restrained to palliative care, but not as disease-modifying drugs. Finally, this study highlighted lentivirus-based gene delivery as an instrumental tool to study the relevance of target genes in synovial joint physiology and disease.

**Electronic supplementary material:**

The online version of this article (doi:10.1186/s13075-017-1326-9) contains supplementary material, which is available to authorized users.

## Background

Rheumatoid arthritis (RA) is a multifactorial autoimmune disease affecting nearly 1% of the world population, whose pathophysiology involves multiple cellular and molecular processes underlying synovial inflammation, joint swelling and pain, and ultimately destruction of articular cartilage and subchondral bone [1]. One of the histopathological hallmarks of RA is a marked increase of mast cell infiltration within the synovium [2–4]. Initial indications on how mast cells could be implicated in the pathophysiology of RA came from studies demonstrating that mast cell-deficient mice, bearing mutations in the c-Kit signalling pathway, developed less severe forms of antigen- or autoantibody-induced arthritis [5–7]. Recent studies, based on more selective strategies for mast cell deficiency in mice, supported a role for these cells in this disease, notably in the pre-clinical phase of T cell-driven antigen-induced arthritis models [8, 9].

The trypsin-like serine protease isoenzymes tryptase-α/β1 (TPSAB1) and -β2 (TPSB2), commonly referred as tryptases, are amongst the most abundant proteases stored within mast cell secretory granules and are pivotal pro-inflammatory mediators in the pathophysiology of allergic and inflammatory diseases [10, 11]. For instance, genetically modified mice lacking mast cell protease-6 and -7 (*Mcpt-6* and *Mcpt-7*), have reduced inflammatory and degenerative parameters associated to experimental arthritis [12, 13]. Indeed, tryptases are emerging as potential therapeutic targets to treat chronic inflammatory diseases. However, these enzymes are stored and secreted as tetramers, wherein the active site is sheltered within the oligomeric catalytic pocket. While this tetrameric assembly conveys substrate specificity, it renders this enzymatic complex resistant to most of the endogenous circulating anti-peptidases, such as α1-anti-trypsin and α2-macroglobulin [14, 15]. Additionally, this structural arrangement challenges the design of highly selective and orally bioavailable inhibitors [16].

Interestingly, the N-terminal region of the recombinant human β-defensin sperm-associated antigen 11B isoform D (hSPAG11B/D), which is conserved in hSPAG11B/C, has been reported in vitro as a potent inhibitor of tryptase-β1 [17]. β-defensins are anti-microbial proteins primarily associated with host defense [18, 19]. Moreover, increasing evidences suggest that β-defensins have immunomodulatory properties and a variety of other non-immunological activities [20, 21]. Indeed, we recently reported that the rat β-defensin SPAG11C, the ortholog of the human SPAG11B isoform C, hSPAG11B/C, is expressed in articular chondrocytes during the male rat embryonic development [22]. However, their potential contribution in controlling inflammatory conditions such as RA remains elusive.

Herein, we hypothesized that an unbalance between tryptases and their endogenous inhibitors, leading to an increased proteolytic activity, is implicated in the pathophysiology of RA. Therefore, we sought to investigate whether this novel endogenous tryptase inhibitor, SPAG11C, is expressed and under regulation during experimentally induced arthritis in the adult mouse knee joint. Considering evidence from studies employing genetically engineered tryptase-deficient mice [12, 13], we also evaluated the potential of tryptase inhibition as a therapeutic alternative for the management of RA. We assessed the impact of two distinct approaches for tryptase inhibition on several inflammatory parameters associated to methylated bovine serum albumin/interleukin-1β (mBSA/IL-1β)-induced arthritis: the lentivirus-mediated heterologous expression of hSPAG11B/C gene product within the adult mouse knee joint, as well as the mouse intra-articular treatment with the synthetic tryptase inhibitor APC366 [23].

## Methods

### Cloning of the hSPAG11B/C coding sequence into pWPXLd-IG

The lentivirus vector pWPXLd (Addgene, Cambridge, MA, USA) was modified by the replacement of the enhanced green fluorescent protein (eGFP) coding sequence by a PCR fragment harboring the internal ribosomal entry site (IRES)-eGFP cassette from pIRES-eGFP vector (Clontech, Mountain View, CA, USA), thus generating the bicistronic vector pWPXLd-IG. Additionally, an adaptor oligonucleotide was inserted between *PmeI* and *BamHI* in order to increase the restriction endonuclease repertoire in the multiple cloning site (*MluI*, *SgfI*, *PvuI*, *RsrII* and *BSu36I*).

The coding sequence of hSPAG11B/C was obtained from human testis and epididymis total ribonucleic acid (RNA) reverse transcribed using ThermoScript RT-PCR System (Thermo Fisher Scientific, Waltham, MA, USA) followed by a PCR performed with the Phusion High-Fidelity DNA Polymerase Kit (NEB, Ipswich, MA, USA) and respective oligonucleotide pair as follows: forward: 5‘-AGTTTAAACGCCACCATGAGGCAACGA-3’; reverse 5’-CTATGGATCCTTAATGTAAACAGCAGGCGTC-3’.

QIAquick purified (Qiagen, GmbH, Hilden, Germany) PCR products were A+ tailed (Thermo Fisher Scientific) and ligated into the transfer plasmid pGEM-T Easy System I (Promega, Madison, WI, USA), transformed into TOP10 competent cells (Thermo Fisher Scientific), amplified and purified using QIAprep Spin Miniprep Kit (Qiagen). Inserts of interest were released from positive pGEM-T plasmids by double digestion with the endonucleases *PmeI* and *BamHI* (NEB), fractioned by preparative agarose gel electrophoresis, purified with the QIAquick Gel Extraction Kit (Qiagen) and ligated into linearized and dephosphorylated (NEB) pWPXLd-IG lentivirus vectors using a 1:3 vector to insert ratio with the T4 DNA Ligase Kit (Thermo Fisher Scientific). For simplification reasons, the resulting vector pWPXLd-hSPAG11B/C-IG will be referred as phSPAG11B/C. NEB 5-alpha electrocompetent *E. coli* were transformed with the ligation reaction mix using a MicroPulser Electroporator (Bio-Rad, Hercules, CA, USA). Clones were amplified and purified and subcloning efficiency was confirmed by automatic DNA sequencing. Lentivirus transfer and the structural vectors pMD2.G and psPAX.2 (Addgene plasmids #12260 and #12259, both provided by D. Trono) were amplified and purified using the NucleoBond® Xtra Maxi Plus EF Kit (Macherey-Nagel, GmbH, Düren, Germany).

### Lentivirus production and titration

HEK293T/17 cells were cultured according to supplier’s recommendations (ATCC, Manassas, VA, USA). Cells (1.7 × 10^7^ per plate) were seeded into 10-cell culture flasks (175 cm^2^) containing 30 mL of DMEM (Gibco, Carlsbad, CA, USA) and then incubated at 37 °C 5% CO_2_. The next day, cells were transfected with a mixture of structural (146 μg of psPAX2 and 79 μg of pMD2.G) and transfer vectors (225 μg of pWPXLd-IG or phSPAG11B/C), by using the transfection reagent GeneJuice (EMD Millipore, Billerica, MA, USA). Cells were incubated overnight at 37 °C 5% CO_2_, then the medium was replaced by 18 mL of OptiMEM (Gibco). Cell culture supernatants were harvested 24 and 48 h later. Each supernatant was cleared by centrifugation and filtration with a 0.45 μm syringe filter and stored at 4 °C. The virus harvests from 24 and 48 h were pooled and layered onto 5 mL of a 20% sucrose solution in Dulbecco's phosphate-buffered saline (DPBS) containing Ca^2+^ and Mg^2+^ and then centrifuged at 106,750 × *g* for 2 h. The pellets were solubilized in DPBS, the samples were fractioned into 20 μL aliquots and stored at -80 °C until use. For the biological titration of the lentiviruses, HEK293T/17 cells (4 × 10^4^ per well) were seeded into a 24-well plate containing coverslips. The next day, cells were transduced with a serial dilution of lentivirus (10^−3^ to 10^−8^) and cultivated for an additional 72 to 92 h. Cells were fixed with 4% buffered formalin and processed for immunofluorescence, as described below. The protocol presented above is the final standardization of several attempts to optimize the production of recombinant lentivirus at high titers for in vivo use.

### Animals

Male C57BL/6 mice (N = 127, 8–10 weeks old, weighting from 22.5–27.3 g), which naturally lack functional *Mcpt-7*, were from Institute of Pharmacology and Molecular Biology (INFAR) animal facility (Universidade Federal de São Paulo – Escola Paulista de Medicina, UNIFESP-EPM, São Paulo, Brazil) and from Janvier Labs (Le Genest-Saint-Isle, France). Mice were housed in groups of up to five animals per cage with access to standard food and water ad libitum, and maintained in level 2 biosafety installations under a 12/12 h light/dark cycle and controlled room temperature (22 ± 1 °C). All invasive or stressful procedures were performed in animals under anaesthesia, either inhalatory isoflurane 1.5% v/v in oxygen or intraperitoneal ketamine (80 mg/kg and xylazine 20 mg/kg, respectively).

### Induction and analysis of experimental arthritis

Animals were submitted to methylated bovine serum albumin/interleukin-1β (mBSA/IL-1β)-induced arthritis, as previously described [12]. Briefly, arthritis was induced by one intra-articular injection in the right knee joint with 10 μL of mBSA (Sigma-Aldrich, St. Louis, MO, USA) at 20 mg/mL in vehicle (PBS; phosphate-buffered saline), followed by three subcutaneous daily injections in the ipsilateral rear footpad with 250 ng of recombinant human interleukin 1β (rhIL-1β; Peprotech, Rocky Hill, NJ, USA) diluted in 20 μL of vehicle (0.9% sodium hydrochloride (NaCl) solution with 0.5% normal C57BL/6 mouse serum). Animals from the control group were submitted to the same procedures, but injected with the respective vehicle solutions. Oedema formation was monitored by measurements (triplicates) of the medio-lateral knee joint diameter by using a specially designed spring-loaded calliper (model #C1X018; Kroeplin, GmbH, Schlüchtern, Germany) on days 0, 1, 2, 5 and 7. Animals were sacrificed at the peak of disease, 7 days after the induction of arthritis [24]. The knee joint samples were harvested and processed accordingly to the downstream applications to be performed. For histopathological analysis, the knee joints were fixed in 4% buffered formaldehyde, decalcified and embedded in paraffin. Medial sections with 5 μm thickness were obtained and submitted to haematoxylin and eosin (H&E) or safranin O staining. The severity of arthritis was scored by observers, single blinded to the experimental groups, by using a semi-quantitative scale based on a previously established method [12, 24, 25]. Briefly, the severity of arthritis in coded slides was graded from 0 (normal) to 5 (severe) for five components that comprised joint space exudate, synovitis, pannus formation, cartilage degradation and bone erosion. Likewise, joint space exudate was identified as leukocytes, either scattered or in aggregates within the joint space. Synovitis was defined as hyperplasia of the synovial membrane due to the proliferation of lining layer fibroblast-like synoviocytes as well as to the infiltration of polymorph and mononuclear leukocytes. Pannus formation was defined as hypertrophic synovial tissue forming tight junctions with articular surfaces. The extent of cartilage and bone erosion was evaluated separately on both condylar surfaces. Scoring was based on the loss of cartilage matrix, disruption and loss of cartilage surface, and the extent and depth of subchondral bone erosion. The average score for two sections analysed from each joint was calculated for each component, then the mean values from the five components was summed, giving an overall mean histopathological severity score for each joint (maximum possible score of 25).

### Lentivirus-mediated heterologous expression of hSPAG11B/C

Animals were submitted to an intra-articular injection into the right knee joint with 20 μL of 2 × 10^6-7^ transduction units (TU) per joint of pWPXLd-IG or phSPAG11B/C. Arthritis was induced 7 days later, as described above. Seven days after the induction of arthritis, the animals were sacrificed; the knee joints were harvested and processed for histopathological analysis as described above. In other sets of experiments, 7 days after the induction of arthritis, the knee joint was washed twice with 25 μL of sterile saline, which was pooled and then centrifuged at 3000 × *g* for 5 min at 4 °C. The resulting cell pellets and supernatants were stored separately at -80 °C until required for downstream experiments. Additionally, the knee joint was harvested and stored at -80 °C. As a control procedure for intra-articular lentivirus injection, in every experimental set, one group of animals was injected with the same volume of lentivirus vehicle (PBS), 7 days prior to the induction of arthritis.

### Intra-articular administration of APC366

Mice were treated by intra-articular injections with the synthetic tryptase inhibitor APC366 (10 μL of 10 or 100 μM; Ki = 7.1 μM) or its vehicle (DMSO 0.1%) 1 h before the induction of arthritis, which were repeated every other day (days 2, 4 and 6). Oedema formation was monitored as described above. The animals were killed 7 days later and both the synovial fluid and knee joints were harvested for downstream assays, as described above.

### Reverse transcription, end-point and semi-quantitative PCR

Total RNA was extracted from knee joints using the RNeasy Mini Plus Kit (Qiagen) and 1 μg was reverse transcribed with the ThermoScript RT-PCR System and Oligo d(T), according to manufacturer’s instructions (Thermo Fisher Scientific). For end-point PCR, complementary deoxyribonucleic acid (cDNA) was amplified with Taq DNA polymerase Kit (Thermo Fisher Scientific) and 800 nM of each forward and reverse oligonucleotide (Table 1). Semi-quantitative PCR (qPCR) was performed with the SYBR Fast q-PCR Kit (Kapa Biosystems, Cape Town, South Africa) and 300 nM of each forward and reverse oligonucleotide (Table 1). The relative expression of the target gene was normalized to the endogenous control *Hprt1*, using the method 2^-DDCt^ [26]. The products from end-point, and in some cases, from semi-quantitative PCR, were loaded onto 2% agarose gels containing ethidium bromide, visualized under UV illumination and photographed.

**Table 1:** List of oligonucleotides

Gene symbol	Strand	Sequence (5’-3’)	bp	Accession number
*SPAG11B/C*	Forward	TGTTTCCAGGATCGTCTC	251	NM_058203
	Reverse	GCCTACTTGTGTTTCCAT		
*Spag11c*	Forward	CTTACCACGAGCCTGAAC	139	NM_001039563
	Reverse	AACGGATGTAAGCAGCAG		
*Spag11a*	Forward	ACAGAGAGCGAGCCGTAAAA	113	NM_153115
	Reverse	AGGCACACGGTGTTTCTGAT		
*Mcpt-6*	Forward	TGAGGCTTCTGAGAGTAA	403	NM_010781
	Reverse	GAGAGGCTCGTCATTATC		
*Hprt1*	Forward	TCCATTCCTATGACTGTAGA	90	NM_013556
	Reverse	ATCATCTCCACCAATAACTT		

Gene symbols, oligonucleotide sequences, predicted base pair (bp) number for amplicons and NCBI accession numbers

### Immunofluorescence

Briefly, after permeabilization in a solution containing PBS, 0.1% Triton X-100 and 1% BSA (Sigma-Aldrich), cells were incubated with a primary antibody anti-GFP raised in goat (1:500, Rockland, Houston, TX, USA), followed by an incubation with a secondary antibody conjugated to AlexaFluor® 555 (1:1000, Invitrogen, Carlsbad, CA, USA). Slides were mounted with Prolong Gold containing 4',6-diamidino-2-phenylindole (DAPI) (Invitrogen). The number of GFP-positive colonies was counted by epifluorescence microscopy and expressed as transduction units (TU) per mL. Knee joint sections with 10 μm thickness were obtained in a cryostat and mounted onto Superfrost slides (Thermo Fisher Scientific). After permeabilization, the sections were incubated with a primary antibody anti-GFP raised in rabbit (1:1000; Invitrogen) overnight at 4 °C, and then processed as described above. Images were acquired using a Zeiss LSM-710 confocal microscope (Carl Zeiss MicroImaging, GmbH, Jena, Germany).

### In situ hybridization assays

In situ hybridization was conducted as described previously [22]. Briefly, formalin-fixed paraffin-embedded tissue sections (4 μm) from knee joints were deparaffinized, rehydrated and treated with proteinase K (10 μg/mL for 10 min, room temperature). Sections were then incubated for 16 h at 37 °C with hybridization buffer (30% formamide, 50 mM Tris-HCl, 5 mM EDTA, 618 mM NaCl, and 10% dextran sulfate) containing 200 nM of locked nucleic acid (LNA)-modified antisense oligonucleotide probes labelled with digoxigenin at their 3′- and 5′-ends (Exiqon, Vedbaek, Denmark). The antisense probe targeting an exon–exon junction of the *Spag11c* transcript sequence was 5′-TGGTCCAGGCTCATGGTAAGG-3′. A scrambled LNA probe was used as a negative control. Sections were washed in series of graded saline-sodium citrate buffer (SSC) solutions (5, 1, and 0.2 × SSC) at 37 °C. Hybridized mRNA was detected using sheep antibody anti-digoxigenin conjugated with horseradish peroxidase (Roche, Indianapolis, IN, USA; 1:50 v/v) and peroxidase activity was revealed by 3,3′-diaminobenzidine (DAB) reaction. Sections were counterstained with Toluidine blue and mounted with Permount (Thermo Fisher Scientific). Slides were visualized with a Nikon E800 microscope (Nikon, Melville, NY, USA) and images acquired using a CoolSNAP-Pro CCD digital camera and Image-Pro Express Software (Media Cybernetics, Silver Spring, MD, USA).

### Myeloperoxidase (MPO) activity

MPO activity was measured as an index of granulocyte infiltration, as previously described [27]. Briefly, cell pellets from knee joint lavages were homogenized in a potassium phosphate-buffered (pH = 6) solution containing 0.5% hexadecyltrimethylammonium bromide. MPO activity was measured in the presence of *o*-dianisidine dihydrocholoride (Sigma-Aldrich) by optical density readings at 450 nm in a FlexStation 3 microplate reader (Molecular Devices, Sunnyvale, CA, USA). Sample results were interpolated into a linear regression generated with a standard curve of MPO (0.05–0.8 U/mL; Sigma-Aldrich). Data were expressed as units of MPO/mL.

### Tryptase-like activity assay

Tryptase-like activity was measured in the supernatant from knee joint lavages in the presence of 200 μM benzyloxycarbonyl-glycine-proline-arginine-7-amino-4-methylcoumarin (Z-GPR-AMC) (Enzo Life Sciences, GmbH, Lörrach, Germany) buffered in 50 mM 4-(2-hydroxyethyl)-1-piperazineethanesulfonic acid (HEPES) and 120 mM NaCl (pH = 8.0) with 365/440 nm (excitation/emission) in a Varioskan Flash microplate reader (Thermo Fisher Scientific). Sample results were interpolated into a linear regression generated with a standard curve of recombinant human tryptase-β1 (0.5–4.0 mU/mL; Promega). Data was expressed as mU of tryptase-like activity per mL.

### Cytokine and chemokine quantification

The concentration of IL-1β, -6, -17A and (C-X-C) ligand (CXCL)1/KC in the supernatant of knee joint washes was determined by using CBA Flex Kits, according to the manufacturer’s instructions (BD, Franklin Lakes, NJ, USA). Data were acquired in a FACSCanto II flow cytometer (BD) and analyzed with the program FCAP Array v3.0 (Soft Flow Inc., Pecs, Hungary). Detection limits for IL-1β, IL-6, IL-17A and CXCL1/KC were 4.32, 9.01, 8.72 and 10.48 pg/mL, respectively.

### Statistical analysis

Data were analysed by using the software Prism version 6 (GraphPad Software, San Diego, CA, USA). Data comparison between the two groups was performed by unpaired Student’s *t* test. The data from the histopathological scores were analysed by Kruskal-Wallis and Dunn’s multiple comparisons test. The time course of oedema formation was analysed by two-way ANOVA and Tukey’s multiple comparisons test. The remaining data was analysed by ordinary one-way ANOVA and Tukey’s multiple comparisons test. Data are expressed as mean ± SEM and values of *P* < 0.05 were considered as statistically significant.

## Results

### The proteolytic balance is impaired in mBSA/IL-1β-induced arthritis

**Fig. 1:**
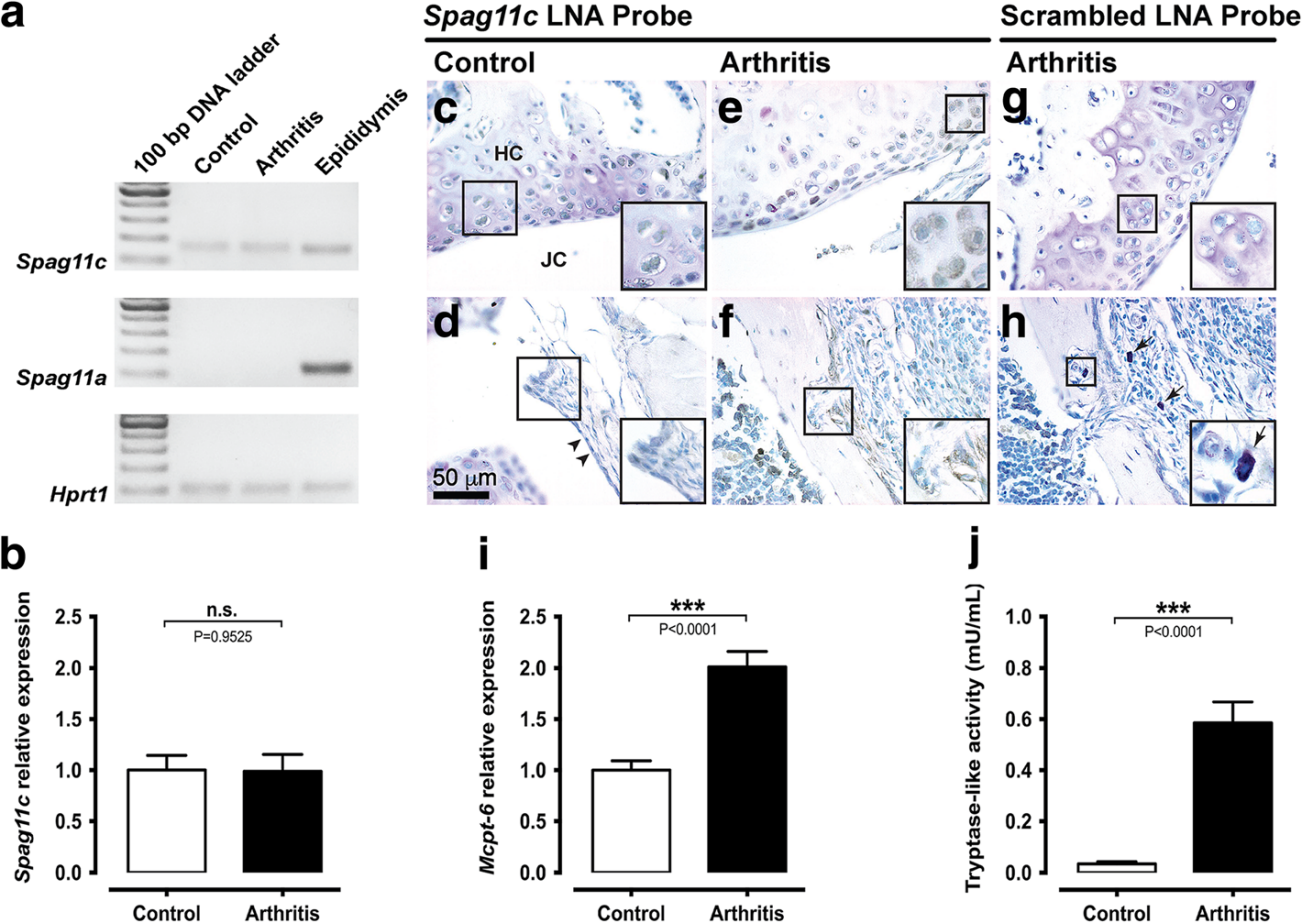
The proteolytic balance is impaired in mBSA/IL-1β-induced arthritis. **a**. Analytical agarose gel electrophoresis of RT-qPCR products for *Spag11c* and *Spag11a* mRNA in knee joints from control or arthritis mice. *Hrpt1* was used as an endogenous control. Adult mouse epididymis cDNA was used as positive control for *Spag11c and Spag11a* mRNA amplification. **b**. RT-qPCR analysis of *Spag11c* relative expression in control and arthritis mouse knee joints. N = 6 mice per group. **c**-**h**. In situ hybridization with *Spag11c* or scrambled digoxigenin-labelled LNA probes in knee joint sections from control and arthritis mice. Hybridization signal was detected in chondrocytes within the hyaline cartilage from control and arthritis mice (**c** and **e**) and in cells from the synovial membrane (**d** and **f**). No staining was observed in negative control assays with scrambled digoxigenin-labelled LNA probe, neither in hyaline cartilage (**g**) nor in the synovial membrane (**h**). *Right bottom insets* represent magnifications of the respective square-demarked areas. The *arrowheads* indicate the normal synovial lining in control animals. The *arrows* evidence the presence of several mast cells in arthritis mice. Images are representative of five mice per group. **i**. RT-qPCR analysis of *Mcpt-6* relative expression in control and arthritis mouse knee joints. N = 11–12 mice per group. **j**. Measurement of tryptase-like activity in the synovial fluid from knee joints of control and arthritis mice. N = 13–15 mice per group. *Abbreviation*: *n.s*. not significant

Transcripts for *Spag11c* were constitutively expressed in the knee joint from control mice, whereas transcripts for *Spag11a* were not detected. On the other hand, both transcripts were detected in positive control samples prepared with RNA extracted from adult mice epididymis (Fig. 1a). Additionally, the relative expression of *Spag11c* did not change 7 days after induction of arthritis (Fig. 1b). In situ hybridization revealed a positive signal for *Spag11c* mRNA in chondrocytes of the hyaline cartilage and in cells from the synovial membrane of the knee joint from control (Fig. 1c and d) and mice submitted to mBSA/IL-1β-induced arthritis (Fig. 1e and f). No hybridization signal was detected in slices processed with a negative control scrambled probe (Fig. 1g and h). By qualitative analysis of knee joint sections stained with Toluidine blue, a few scattered mast cells were observed in the synovia of control mice, whereas 7 days after induction of arthritis, the number of mast cells seemed to increase (Fig. 1h). Accordingly, the relative expression of *Mcpt-6* in the mouse knee joint increased up to twofold 7 days after induction of arthritis (Fig. 1i). Additionally, tryptase-like activity was upregulated in the synovial fluid from the knee joint of mice submitted to mBSA/IL-1β-induced arthritis (Fig. 1j).

### The lentivirus system promotes a stable heterologous expression of hSPAG11B/C

**Fig. 2:**
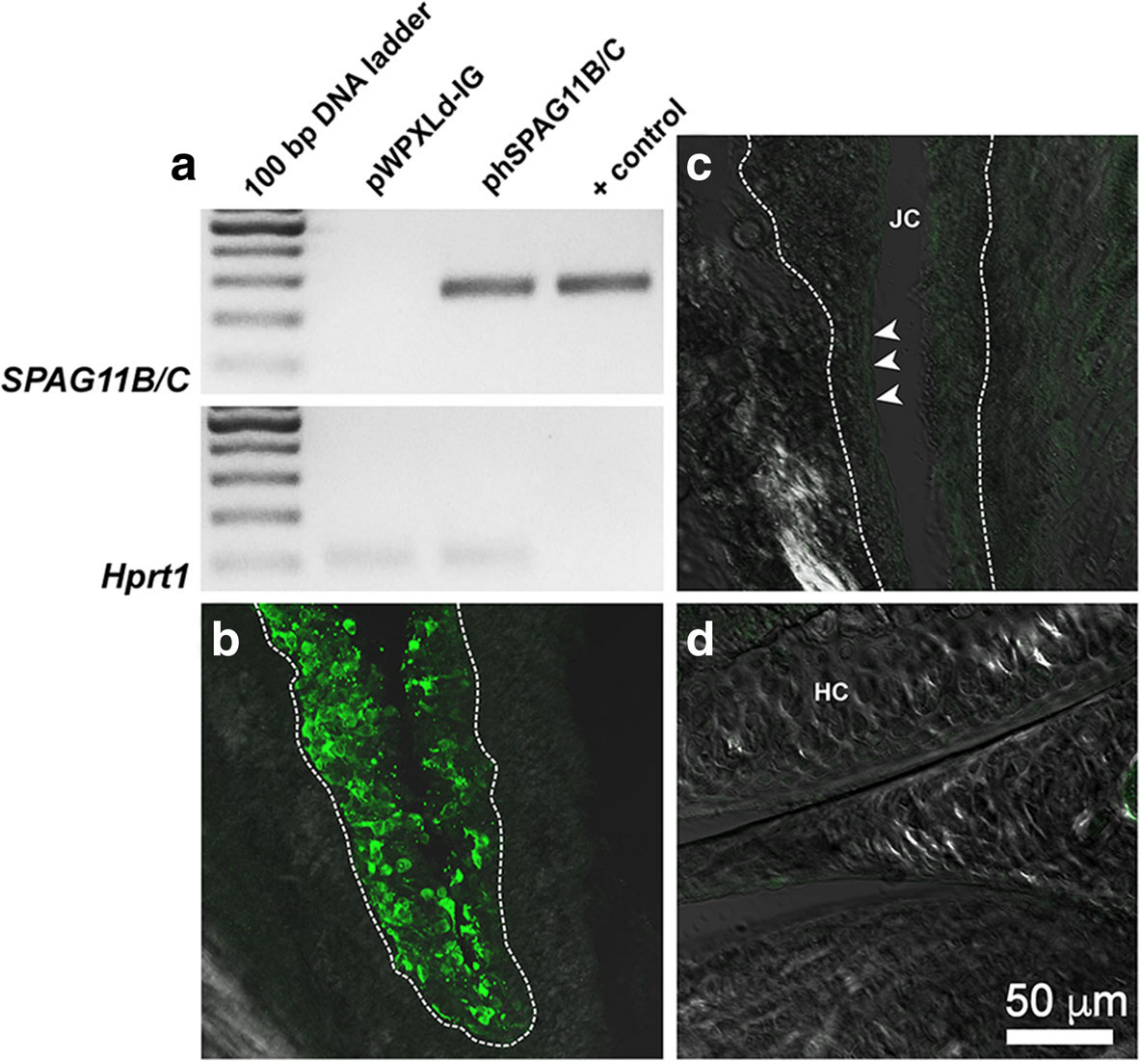
The lentivirus system promoted a stable heterologous expression of hSPAG11B/C. **a**. Representative agarose-gel electrophoresis of end-point RT-PCR for *SPAG11B/C* in knee joints transduced with pWPXLd-IG or phSPAG11B/C from animals killed 7 days after induction of arthritis. The lentivirus vector phSPAG11B/C (1 × 10^3^ copies) was used as DNA template for the indicated positive control (+ control). *Hrpt1* was used as an endogenous control. **b**-**d**. Representative confocal photomicrographs of GFP immunoreactivity (pseudocolored in *green*) performed in knee joints, evidencing the synovial membrane from a mouse transduced with 1 × 10^7^ TU/joint of phSPAG11B (**b**), the synovial membrane of a mouse not transduced with lentivirus (**c**) and the hyaline cartilage from a mouse transduced with 1 × 10^7^ TU/joint of phSPAG11B (**d**). The *dashed line* encircles the inner boundaries of the synovia and the *arrowheads* indicate the lining layer of the synovial membrane. *Abbreviations*: *JC* joint cavity, *HC* hyaline cartilage

The protocol for lentivirus production presented in this study provided consistent titers ranging from 1.1 × 10^9^ up to 2.0 × 10^10^ TU/mL. We then evaluated if transgene expression was still detected in the knee joint up to 7 days after induction of inflammatory arthritis. Indeed, the *SPAG11B/C* transcript was detected in knee joint samples from mice previously injected with the corresponding lentivirus phSPAG11B/C (Fig. 2a). Additionally, immunofluorescence studies demonstrated that the transgene integration occurred predominantly in synoviocytes, since GFP-positive cells were restricted to the synovial membrane (Fig. 2b and c), while no positive cells were observed in the cartilage (Fig. 2d).

### Lentivirus-mediated heterologous expression of hSPAG11B/C or treatment with APC366 re-establishes the proteolytic balance

**Fig. 3:**
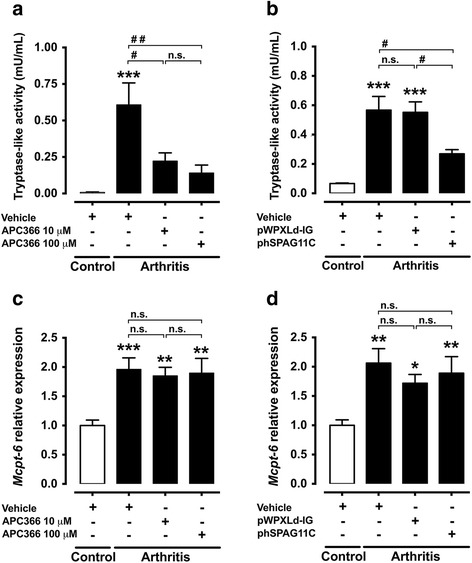
Effect of lentivirus-mediated heterologous expression of hSPAG11B/C or APC366 on proteolytic balance. **a** and **b**. Effect of the synthetic tryptase inhibitor APC366 (10–100 μM) or the transduction with the lentiviruses phSPAG11B/C or pWPXLd-IG, as control, in the tryptase-like activity in the synovial fluid of mouse knee joints 7 days after the induction of arthritis. ^***^ *P* < 0.001 vs. control group; ^#^ *P* < 0.05; ^##^ *P* < 0.01 vs. arthritis groups; n = 6–8 per group). **c** and **d**. Relative expression of *Mcpt-6* in the mouse knee joint 7 days after induction of arthritis in animals treated with APC366 or transduced with phSPAG11B/C or pWPXLd-IG. ^*^ *P* < 0.05; ^**^ *P* < 0.01; ^***^ *P* < 0.001 vs. control groups (n = 5–6 per group). *Abbreviation*: *n.s.* not significant

The *Mcpt-6* transcript level and tryptase-like activity in the synovial fluid were both increased in the mouse knee joint 7 days after the induction of arthritis (Fig. 3). The intra-articular injection of the tryptase inhibitor APC366 (10–100 μM) or the transduction of knee joints with phSPAG11B/C reduced the tryptase-like activity in animals with arthritis. In contrast, transduction with the control lentivirus pWPXLd-IG or injection with vehicle had no effect (Fig. 3a and b). Neither APC366 nor transduction with phSPAG11B/C, however, reverted the increase of *Mcpt-6* gene expression observed 7 days after induction of arthritis (Fig. 3c and d).

### Lentivirus-mediated heterologous expression of hSPAG11B/C or treatment with APC366 has marginal impact on mBSA/IL-β-induced arthritis

**Fig. 4:**
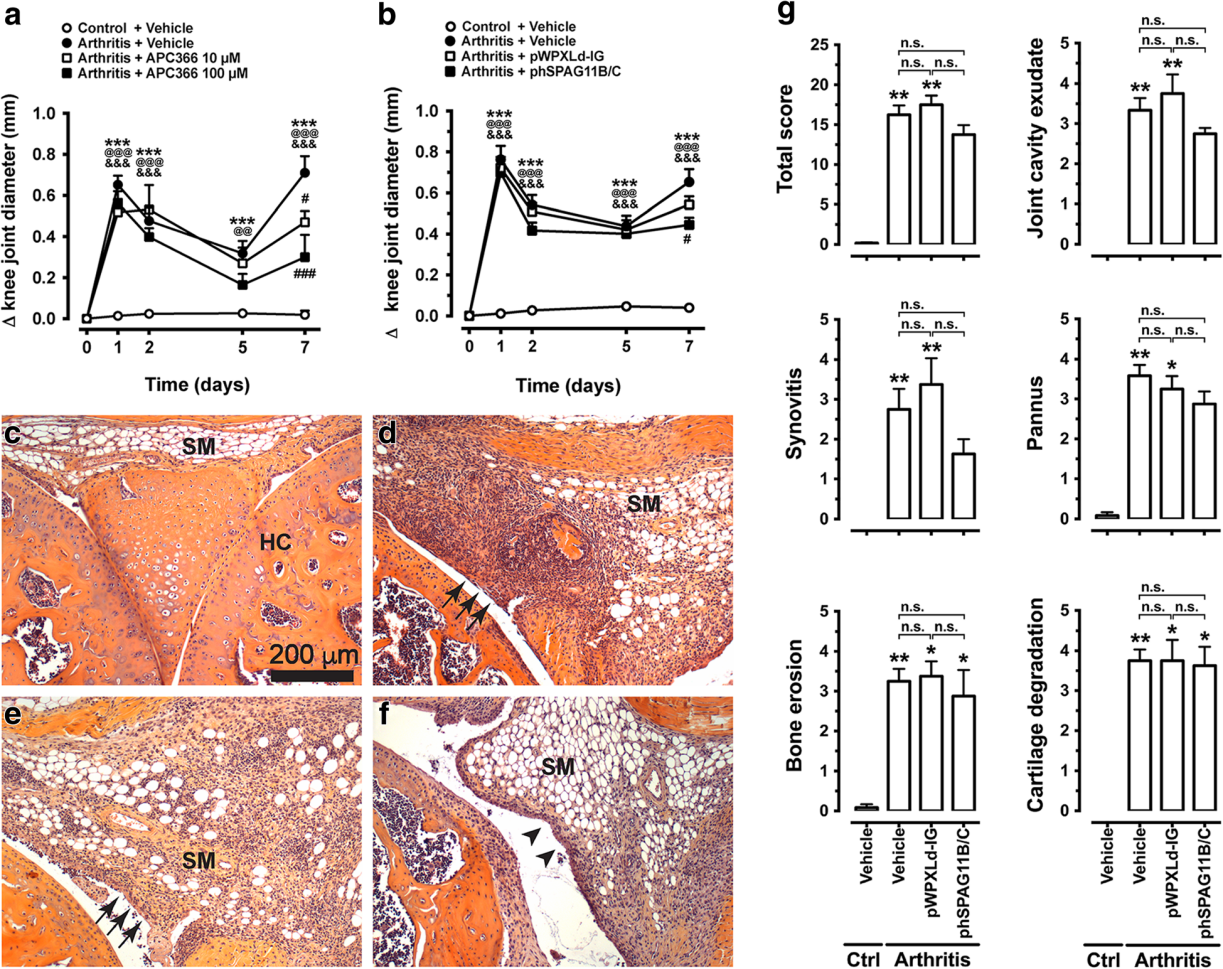
Effect of lentivirus-mediated heterologous expression of hSPAG11B/C and APC366 in mBSA/IL-β-induced arthritis. **a** and **b**. Time course of oedema formation (increase in knee joint medio-lateral diameter) after induction of arthritis with mBSA/IL-1β in animals treated with the synthetic tryptase inhibitor APC366 at 10–100 μM (N = 6–8 mice per group), or previously transduced with pWPXLd-IG or phSPAG11B/C (N = 13–14 mice per group). ^***^ *P* < 0.001, arthritis + vehicle groups vs*.* control; ^@@^ *P* < 0.01; ^@@@^ *P* < 0.001, arthritis + APC366 (10 μM) or arthritis + pWPXLd-IG groups vs*.* control; ^&&&^ *P* < 0.001, arthritis + APC366 (100 μM) or arthritis + phSPAG11B/C groups vs*.* control; ^#^ *P* < 0.05; ^###^ *P* < 0.001, arthritis + APC366 (100 μM) or arthritis + phSPAG11B/C groups vs*.* arthritis + vehicle. **c**-**f**. Representative light photomicrographs of knee joint slices stained by the method of eosin and haematoxylin from a control animal (**c**), or animals submitted to mBSA/IL-1β-induced arthritis, previously injected with vehicle (**d**), or transduced with 2 × 10^6^ TU/joint of pWPXLD-IG (**e**) or phSPAG11B/C (**f**). The *arrows* indicate areas of intense synovitis. The *arrowheads* show areas with moderate synovitis. **g**. Total and individual histopathological scores of inflammatory parameters (N = 4–6 mice per group). ^*^ *P* < 0.05; ^**^ *P* < 0.01; ^***^ *P* < 0.001, vs*.* control group. *Abbreviations*: *JC* joint cavity, *SM* synovial membrane, *Ctrl* control, *n.s*. not significant

Animals submitted to mBSA/IL-1β-induced arthritis presented a long-lasting and biphasic oedema, which peaked on days 1 and 7. The intra-articular treatment with APC366 (10–100 μM) reduced the late phase of oedema formation, 7 days after the induction of arthritis (Fig. 4a). Similarly, animals with arthritis previously transduced with phSPAG11B/C, but not with pWPXLd-IG, presented reduced oedema at day 7 (Fig. 4b).

Histopathological analysis evidenced that while knee joints from control animals presented no signs of joint inflammation (Fig. 4c), mBSA/IL-1β-treated mice developed a severe arthritis 7 days after its induction, with an intense influx of leukocytes to the knee joint cavity and synovial membrane (synovitis), pannus formation, subchondral bone erosion (Fig. 4d) and degradation of the extracellular matrix of hyaline cartilage (Additional file 1). Similarly, animals previously transduced with pWPXLd-IG developed severe arthritis (Fig. 4e). The knee joints of mice transduced with phSPAG11B/C presented a trend for a lower degree of inflammatory arthritis, particularly regarding synovitis and joint cavity exudate (Fig. 4f). Although none of the histopathological parameters was significantly reduced in comparison to animals submitted to mBSA/IL-1β-induced arthritis, either pre-injected with vehicle or transduced with the control lentivirus pWPXLd-IG (Fig. 4g).

**Fig. 5:**
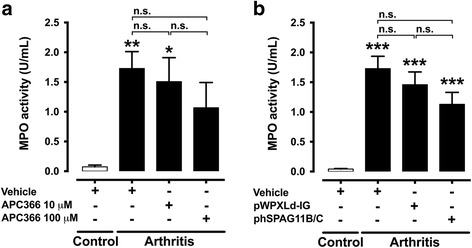
Effect of lentivirus-mediated heterologous expression of hSPAG11B/C and APC366 on MPO activity. **a** and **b**. MPO activity in the synovial fluid of knee joint 7 days after induction of arthritis in animals treated with APC366 at 10–100 μM (N = 6–8 mice per group) or previously transduced with pWPXLd-IG or phSPAG11B/C (N = 14–15 mice per group). ^*^ *P* < 0.05; ^**^ *P* < 0.01; ^***^ *P* < 0.001 vs. control groups. *Abbreviation*: *n.s*. not significant

MPO activity, a biochemical index of granulocyte infiltration, increased in the joint cavity exudate 7 days after induction of arthritis, when compared to control animals. The treatment of animals with APC366 or the transduction with phSPAG11B/C had no major effect on the increased MPO activity (Fig. 5a and b).

### Lentivirus-mediated heterologous expression of hSPAG11B/C or treatment with APC366 inhibits the production of inflammatory mediators

**Fig. 6:**
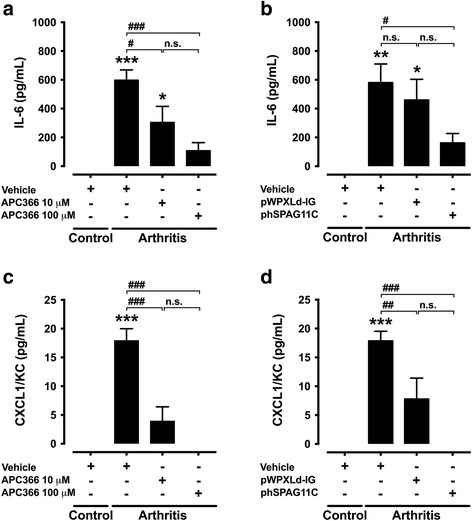
Effect of lentivirus-mediated heterologous expression of hSPAG11B/C and APC366 on cytokines and chemokine production. **a** and **b**. Effect of the synthetic tryptase inhibitor APC366 (10–100 μM) or the knee joint transduction (1 × 10^7^ TU/joint) with phSPAG11B/C in the release of IL-6 (**a** and **b**) and CXCL1/KC (**c** and **d**) 7 days after the induction of arthritis with mBSA/IL-1β. (N = 6 mice per group). ^*^ *P* < 0.05; ^**^ *P* < 0.01; ^***^ *P* < 0.001 vs. control groups; ^#^ *P* < 0.05; ^##^ *P* < 0.01; ^###^ *P* < 0.001 vs*.* arthritis + vehicle groups. *Abbreviation*: *n.s.* not significant

The release of IL-6 and CXCL1/KC to the synovial fluid increased 7 days after induction of arthritis, *albeit* CXCL1/KC concentration was just slightly above the detection limit (Fig. 6). This increase of IL-6 associated to mBSA/IL-1β-induced arthritis was dose-dependently reduced by the treatment with APC366 (Fig. 6a), or the previous transduction with phSPAG11B/C (Fig. 6b). The control lentivirus pWPXLd-IG had no effect on IL-6 release. Similarly, the production of CXCL1/KC was reduced by the treatment with APC366 (Fig. 6c), or the previous transduction with phSPAG11B/C (Fig. 6d). However, the transduction with pWPXLd-IG also reduced the production of CXCL1/KC. Neither IL-1β nor IL-17A was detected in any of the experimental conditions tested.

## Discussion

Previous studies have shown that rheumatoid arthritis and experimental models of this disease are associated with an intense mastocytosis and mast cell degranulation in affected joints, suggesting that an increased release of active tryptase occurs in this disease [2–4, 12]. Since proteolytic activity can be counterbalanced by the release of endogenous inhibitors, we initially evaluated if an actual increased proteolytic activity due to an unbalance between mast cell-restricted tryptase and endogenous inhibitors takes place during joint inflammation. Indeed, this hypothesis was supported by our results, wherein *Mcpt-6* gene expression as well as tryptase-like activity were upregulated 7 days after mBSA/IL-1β-induced arthritis to levels seemingly not counterbalanced by endogenous tryptase inhibitors, such as SPAG11B/C. However, in animals subjected to lentivirus-mediated heterologous expression of SPAG11B/C, as well as to the treatment with APC366, the proteolytic balance was re-established to a significant extent, since both strategies inhibited the increase of tryptase-like activity in the synovial fluid, 7 days after induction of arthritis. In contrast, neither the recombinant expression of SPAG11B/C nor the treatment with APC366 impacted the upregulation of *Mcpt-6* gene expression, thus indicating that the reduced tryptase-like activity was due to enzymatic inhibition. Hence, in view of the upregulated tryptase-like activity, we investigated whether the re-establishment of the proteolytic balance by tryptase inhibition would be beneficial.

Initially, we assessed the feasibility of using a lentivirus-mediated expression system to promote the stable expression of the β-defensin SPAG11B/C in synovial joints. It is worthwhile mentioning that the protocol for lentivirus production presented herein yielded the production of constant high titers of functional lentiviral particles. Likewise, in transduced animals, both GFP and SPAG11B/C were still detected 14 days after mice knee joint transduction, indicating a stable expression of transgenes, even after the induction of arthritis, wherein an extensive hyperplasia of fibroblast-like synoviocytes occurred. Additionally, GFP-positive cells were restricted to the synovial membrane, whereas no positive cell was observed in chondrocytes, in accordance with previous studies using VSV-G pseudotyped HIV-1 derived lentivirus in knee joints of rodents [28, 29]. Furthermore, a previous study reported the occurrence of only negligible levels of transduction of off-target organs after an intra-articular injection of lentiviral particles in synovial joints [28]. As a whole, this indicates that lentivirus-based gene delivery is an instrumental tool to study the relevance of target genes in synovial joint physiology and disease.

We then screened the anti-inflammatory potential of SPAG11B/C isoform by using the lentivirus-mediated expression system, in parallel to the treatment with the synthetic tryptase inhibitor APC366. Mast cell-restricted tryptases are well-established oedematogenic mediators, by a mechanism involving activation of PAR2 receptors [30–32]. For instance, an intra-articular injection of synthetic hexapeptides corresponding to the tethered ligand sequence revealed after PAR2 cleavage by trypsin-like serine proteases, such as tryptase, induced swelling in synovial joints from normal rats [33–35]. Besides, a recent study shown that an intra-articular injection of mast cell tryptase into the mouse knee joint induces hyperaemia, oedema and pain [36]. This suggests that an oedematogenic pathway triggered by PAR2 activation is ready to take place in normal tissue conditions. Herein, both strategies of tryptase inhibition reduced oedema formation, but only in the late phase of mBSA/IL-1β-induced arthritis, thus indicating that mast cell accumulation and/or *Mcpt-6* upregulation occur late in this model. Accordingly, the relative level of *Mcpt-6* mRNA was upregulated 7 days after induction of arthritis. As a whole, while previous studies developed by us and others have shown that PAR2 pro-inflammatory and nociceptive signalling pathways are ready to be pharmacologically triggered even in synovial joints from healthy animals, the present study indicates that its endogenous proteolytic activator (i.e. mast cell-restricted tryptase), only becomes quantitatively relevant on the late phase of mBSA/IL-1β-induced arthritis.

Earlier studies based on mast cell-depleted mice due to c-kit signalling deficiency reported a central role of these cells in neutrophil infiltration and joint degeneration [5–7]. Later on it was shown that c-kit deficiency was associated with important hematopoietic impairments, including a high degree of neutropenia, indicating that the resistance of these mice to experimental arthritis was mostly associated to this hematopoietic disorder [37], since neutrophils are pivotal cellular effectors in joint degeneration [38]. Considering that mast cells produce a large repertoire of inflammatory mediators, the role of tryptase in experimental arthritis was investigated in mice lacking *Mcpt-6* and *-7.* This study reported a reduced neutrophil infiltration and joint degeneration associated to mBSA/IL-1β-induced arthritis [12]. Later on, the same group demonstrated that the in vitro stimulation of mouse or human fibroblast-like synoviocytes with tryptase upregulated the expression of the neutrophil chemokines CXCL1/KC, CXCL5/LIX and CXCL8/IL-8 [13, 39]. Similarly, our study demonstrated that the concentration of CXCL1/KC in the knee joint synovial fluid was reduced after tryptase inhibition.

In view of the findings reported with *Mcpt-6* and *-*7 knockout mice [12, 13], we then assessed whether tryptase inhibition would suffice to impact neutrophil infiltration and joint degeneration. As revealed by histopathological analysis, animals submitted to mBSA/IL-1β-induced arthritis, either pre-injected with vehicle or transduced with the control lentivirus pWPXLd-IG, developed a severe inflammatory arthritis, notably with an intense infiltration of leukocytes within the joint cavity and synovial membrane. Surprisingly, although the transduction with phSPAG11B/C or the treatment with APC366 clearly inhibited tryptase-like activity by a significant extent, both inhibitory strategies had only marginal, if any effect on neutrophil infiltration, as depicted by histopathological analysis and MPO activity. This suggests that residual tryptase activity over the baseline level may be sufficient to support neutrophil influx, by a mechanism yet to be identified, but seemingly independent of CXCL1/KC, since the concentration level of this chemokine barely surpassed the detection limits 7 days after the induction of arthritis.

The production of IL-6 is increased in RA [40] and its signalling plays a central role in joint inflammation and degeneration [41]. Indeed, studies with monoclonal antibodies designed to target IL-6 receptors (IL-6R) reported an attenuation of collagen-induced arthritis in mice [42], as well as in human RA [43]. In our study, IL-6 production was upregulated in the synovial fluid of mice 7 days after induction of arthritis. Additionally, IL-6 overproduction was consistently reversed by the recombinant expression of SPAG11B/C or the treatment with APC366. Similarly, a recent study based on collagen-induced arthritis reported a reduction of IL-6 serum levels in mast cell-deficient mice [9]. In this way, our data clearly indicates that tryptase must be the major mast cell mediator regulating IL-6 production in arthritis. Intriguingly, while some inflammatory parameters associated to mBSA/IL-1β-induced arthritis were attenuated by both strategies of tryptase inhibition, the degenerative parameters investigated were not affected. Similarly, a previous study demonstrated that IL-6 knockout mice were only mildly protected from mBSA/IL-1β-induced arthritis [44]. This dichotomy seems to be due to the fact that while IL-6 exert pro-inflammatory actions through activation of soluble IL-6 receptors (sIL-6R), the activation of membrane-bound IL-6 receptors (mIL-6R) is rather protective [41]. In this way, while sIL-6R blockade is beneficial, the global inhibition of IL-6 seems to be detrimental. This may explain the limited anti-inflammatory effect of tryptase inhibition on mBSA/IL-1β-induced arthritis observed in the present study.

## Conclusions

Our study demonstrated that the lentivirus-mediated heterologous expression of an endogenous tryptase inhibitor (hSPAG11B/C) as well as the intra-articular administration of APC366 presented closely overlapping inhibitory effects on tryptase-like activity, oedema formation, IL-6 and CXCL1/KC production, whereas leukocyte infiltration, cartilage degradation and subchondral bone erosion were not affected. These results show that tryptase-β inhibition offers limited effect on mBSA/IL-β-induced arthritis, thus suggesting that the therapeutic application of these inhibitors to rheumatoid arthritis would be restrained to palliative care, but not as disease-modifying drugs, unless the development of far more potent molecules in the future uncovers a broader potential, as previously highlighted by studies based on transgenic animals. Furthermore, clinical relevance may also lay in their potential effectiveness as RA adjuvant therapy if associated with drugs encompassing other inflammatory pathways implicated in the pathophysiology of this disease, a topic that will require future investigation.

## Additional file

Additional file 1: Effect of lentivirus-mediated heterologous expression of hSPAG11B/C and APC366 in mBSA/IL-β-induced arthritis. A-D. Representative light photomicrographs of knee joint sections stained by the method of safranin O from a control animal (A), or animals submitted to mBSA/IL-1β-induced arthritis, previously injected with vehicle (B), transduced with 2 × 10^6^ TU/joint of pWPXLD-IG (C) or phSPAG11B/C (D). The *arrows* indicate areas of intense extracellular matrix degradation in the hyaline cartilage. The *arrowheads* show areas of normal hyaline cartilage. *Abbreviations*: *JC* joint cavity. (TIF 2265 kb)

## Abbreviations

cDNA: Complementary deoxyribonucleic acid
CXCL: (C-X-C) ligand
DAB: 3,3’-diaminobenzidine
DAPI: 4',6-diamidino-2-phenylindole
DPBS: Dulbecco’s phosphate-buffered saline
eGFP: Enhanced green fluorescent protein
HEPES: 4-(2-hydroxyethyl)-1-piperazineethanesulfonic acid
IL: Interleukin
IRES: Internal ribosomal entry site
LNA: Locked nucleic acid
mBSA: Methylated bovine serum albumin
Mcpt-6: Mouse mast cell protease-6
MPO: Myeloperoxidase
n.s: Not significant
NaCl: Sodium hydrochloride
PBS: Phosphate-buffered saline
RA: Rheumatoid arthritis
RNA: Ribonucleic acid
RT-PCR: Reverse transcription-polymerase chain reaction
SPAG11: Sperm-associated antigen 11
SSC: Saline-sodium citrate buffer
TPSAB1: Tryptase-α/β1
TPSB2: Tryptase-β2
Z-GPR-AMC: Benzyloxycarbonyl-glycine-proline-arginine-7-amino-4-methylcoumarin

## References

[1] Lee DM, Weinblatt ME (2001). Rheumatoid arthritis. Lancet.

[2] Crisp AJ, Chapman CM, Kirkham SE, Schiller AL, Krane SM (1984). Articular mastocytosis in rheumatoid arthritis. Arthritis Rheum.

[3] Godfrey HP, Ilardi C, Engber W, Graziano FM (1984). Quantitation of human synovial mast cells in rheumatoid arthritis and other rheumatic diseases. Arthritis Rheum.

[4] Nigrovic PA, Lee DM (2007). Synovial mast cells: role in acute and chronic arthritis. Immunol Rev.

[5] van den Broek MF, van den Berg WB, van de Putte LB (1988). The role of mast cells in antigen induced arthritis in mice. J Rheumatol.

[6] Corr M, Crain B (2002). The role of FcgammaR signaling in the K/B x N serum transfer model of arthritis. J Immunol.

[7] Lee DM, Friend DS, Gurish MF, Benoist C, Mathis D, Brenner MB (2002). Mast cells: a cellular link between autoantibodies and inflammatory arthritis. Science.

[8] Schubert N, Dudeck J, Liu P, Karutz A, Speier S, Maurer M, Tuckermann J, Dudeck A (2015). Mast cell promotion of T cell-driven antigen-induced arthritis despite being dispensable for antibody-induced arthritis in which T cells are bypassed. Arthritis Rheumatol.

[9] van der Velden D, Lagraauw HM, Wezel A, Launay P, Kuiper J, Huizinga TW, Toes RE, Bot I, Stoop JN (2016). Mast cell depletion in the preclinical phase of collagen-induced arthritis reduces clinical outcome by lowering the inflammatory cytokine profile. Arthritis Res Ther.

[10] Stevens RL, Adachi R (2007). Protease-proteoglycan complexes of mouse and human mast cells and importance of their beta-tryptase-heparin complexes in inflammation and innate immunity. Immunol Rev.

[11] Pejler G, Ronnberg E, Waern I, Wernersson S (2010). Mast cell proteases: multifaceted regulators of inflammatory disease. Blood.

[12] McNeil HP, Shin K, Campbell IK, Wicks IP, Adachi R, Lee DM, Stevens RL (2008). The mouse mast cell-restricted tetramer-forming tryptases mouse mast cell protease 6 and mouse mast cell protease 7 are critical mediators in inflammatory arthritis. Arthritis Rheum.

[13] Shin K, Nigrovic PA, Crish J, Boilard E, McNeil HP, Larabee KS, Adachi R, Gurish MF, Gobezie R, Stevens RL (2009). Mast cells contribute to autoimmune inflammatory arthritis via their tryptase/heparin complexes. J Immunol.

[14] Pereira PJ, Bergner A, Macedo-Ribeiro S, Huber R, Matschiner G, Fritz H, Sommerhoff CP, Bode W (1998). Human beta-tryptase is a ring-like tetramer with active sites facing a central pore. Nature.

[15] Caughey GH (2007). Mast cell tryptases and chymases in inflammation and host defense. Immunol Rev.

[16] Cairns JA (2005). Inhibitors of mast cell tryptase beta as therapeutics for the treatment of asthma and inflammatory disorders. Pulm Pharmacol Ther.

[17] Radhakrishnan Y, Hamil KG, Tan JA, Grossman G, Petrusz P, Hall SH, French FS (2009). Novel partners of SPAG11B isoform D in the human male reproductive tract. Biol Reprod.

[18] Selsted ME, Harwig SS, Ganz T, Schilling JW, Lehrer RI (1985). Primary structures of three human neutrophil defensins. J Clin Invest.

[19] Taylor K, Clarke DJ, McCullough B, Chin W, Seo E, Yang D, Oppenheim J, Uhrin D, Govan JR, Campopiano DJ (2008). Analysis and separation of residues important for the chemoattractant and antimicrobial activities of beta-defensin 3. J Biol Chem.

[20] Patil AA, Cai Y, Sang Y, Blecha F, Zhang G (2005). Cross-species analysis of the mammalian beta-defensin gene family: presence of syntenic gene clusters and preferential expression in the male reproductive tract. Physiol Genomics.

[21] Hall SH, Yenugu S, Radhakrishnan Y, Avellar MC, Petrusz P, French FS (2007). Characterization and functions of beta defensins in the epididymis. Asian J Androl.

[22] Ribeiro CM, Queiroz DB, Patrao MT, Denadai-Souza A, Romano RM, Silva EJ, Avellar MC (2015). Dynamic changes in the spatio-temporal expression of the beta-defensin SPAG11C in the developing rat epididymis and its regulation by androgens. Mol Cell Endocrinol.

[23] Krishna MT, Chauhan A, Little L, Sampson K, Hawksworth R, Mant T, Djukanovic R, Lee T, Holgate S (2001). Inhibition of mast cell tryptase by inhaled APC 366 attenuates allergen-induced late-phase airway obstruction in asthma. J Allergy Clin Immunol.

[24] Staite ND, Richard KA, Aspar DG, Franz KA, Galinet LA, Dunn CJ (1990). Induction of an acute erosive monarticular arthritis in mice by interleukin-1 and methylated bovine serum albumin. Arthritis Rheum.

[25] Lawlor KE, Campbell IK, O'Donnell K, Wu L, Wicks IP (2001). Molecular and cellular mediators of interleukin-1-dependent acute inflammatory arthritis. Arthritis Rheum.

[26] Livak KJ, Schmittgen TD (2001). Analysis of relative gene expression data using real-time quantitative PCR and the 2(-Delta Delta C(T)) Method. Methods.

[27] Denadai-Souza A, Camargo Lde L, Ribela MT, Keeble JE, Costa SK, Muscara MN (2009). Participation of peripheral tachykinin NK1 receptors in the carrageenan-induced inflammation of the rat temporomandibular joint. Eur J Pain.

[28] Gouze E, Pawliuk R, Pilapil C, Gouze JN, Fleet C, Palmer GD, Evans CH, Leboulch P, Ghivizzani SC (2002). In vivo gene delivery to synovium by lentiviral vectors. Mol Ther.

[29] Geurts J, Vermeij EA, Pohlers D, Arntz OJ, Kinne RW, van den Berg WB, van de Loo FA (2011). A novel Saa3-promoter reporter distinguishes inflammatory subtypes in experimental arthritis and human synovial fibroblasts. Ann Rheum Dis.

[30] Steinhoff M, Vergnolle N, Young SH, Tognetto M, Amadesi S, Ennes HS, Trevisani M, Hollenberg MD, Wallace JL, Caughey GH (2000). Agonists of proteinase-activated receptor 2 induce inflammation by a neurogenic mechanism. Nat Med.

[31] Palmer HS, Kelso EB, Lockhart JC, Sommerhoff CP, Plevin R, Goh FG, Ferrell WR (2007). Protease-activated receptor 2 mediates the proinflammatory effects of synovial mast cells. Arthritis Rheum.

[32] Lohman RJ, Cotterell AJ, Barry GD, Liu L, Suen JY, Vesey DA, Fairlie DP (2012). An antagonist of human protease activated receptor-2 attenuates PAR2 signaling, macrophage activation, mast cell degranulation, and collagen-induced arthritis in rats. FASEB J.

[33] Denadai-Souza A, Cenac N, Casatti CA, Camara PR, Yshii LM, Costa SK, Vergnolle N, Muscara MN (2010). PAR(2) and temporomandibular joint inflammation in the rat. J Dent Res.

[34] Denadai-Souza A, Martin L, de Paula MA, de Avellar MC, Muscara MN, Vergnolle N, Cenac N (2012). Role of transient receptor potential vanilloid 4 in rat joint inflammation. Arthritis Rheum.

[35] Russell FA, Schuelert N, Veldhoen VE, Hollenberg MD, McDougall JJ (2012). Activation of PAR(2) receptors sensitizes primary afferents and causes leukocyte rolling and adherence in the rat knee joint. Br J Pharmacol.

[36] Borbely E, Sandor K, Markovics A, Kemeny A, Pinter E, Szolcsanyi J, Quinn JP, McDougall JJ, Helyes Z (2016). Role of capsaicin-sensitive nerves and tachykinins in mast cell tryptase-induced inflammation of murine knees. Inflamm Res.

[37] Zhou JS, Xing W, Friend DS, Austen KF, Katz HR (2007). Mast cell deficiency in Kit(W-sh) mice does not impair antibody-mediated arthritis. J Exp Med.

[38] Kaplan MJ (2013). Role of neutrophils in systemic autoimmune diseases. Arthritis Res Ther.

[39] Nakano S, Mishiro T, Takahara S, Yokoi H, Hamada D, Yukata K, Takata Y, Goto T, Egawa H, Yasuoka S (2007). Distinct expression of mast cell tryptase and protease activated receptor-2 in synovia of rheumatoid arthritis and osteoarthritis. Clin Rheumatol.

[40] Hirano T, Matsuda T, Turner M, Miyasaka N, Buchan G, Tang B, Sato K, Shimizu M, Maini R, Feldmann M (1988). Excessive production of interleukin 6/B cell stimulatory factor-2 in rheumatoid arthritis. Eur J Immunol.

[41] Calabrese LH, Rose-John S (2014). IL-6 biology: implications for clinical targeting in rheumatic disease. Nat Rev Rheumatol.

[42] Fujimoto M, Serada S, Mihara M, Uchiyama Y, Yoshida H, Koike N, Ohsugi Y, Nishikawa T, Ripley B, Kimura A (2008). Interleukin-6 blockade suppresses autoimmune arthritis in mice by the inhibition of inflammatory Th17 responses. Arthritis Rheum.

[43] Murakami M, Nishimoto N (2011). The value of blocking IL-6 outside of rheumatoid arthritis: current perspective. Curr Opin Rheumatol.

[44] Lawlor KE, Wong PK, Campbell IK, van Rooijen N, Wicks IP (2005). Acute CD4+ T lymphocyte-dependent interleukin-1-driven arthritis selectively requires interleukin-2 and interleukin-4, joint macrophages, granulocyte-macrophage colony-stimulating factor, interleukin-6, and leukemia inhibitory factor. Arthritis Rheum.

