# Supplementary figures and images for: Effect of tryptase inhibition on joint inflammation: a pharmacological and lentivirus-mediated gene transfer study
Source: Arthritis Res Ther. 2017 Jun 6;19:124. doi: 10.1186/s13075-017-1326-9 (PMC5461776; doi:10.1186/s13075-017-1326-9)

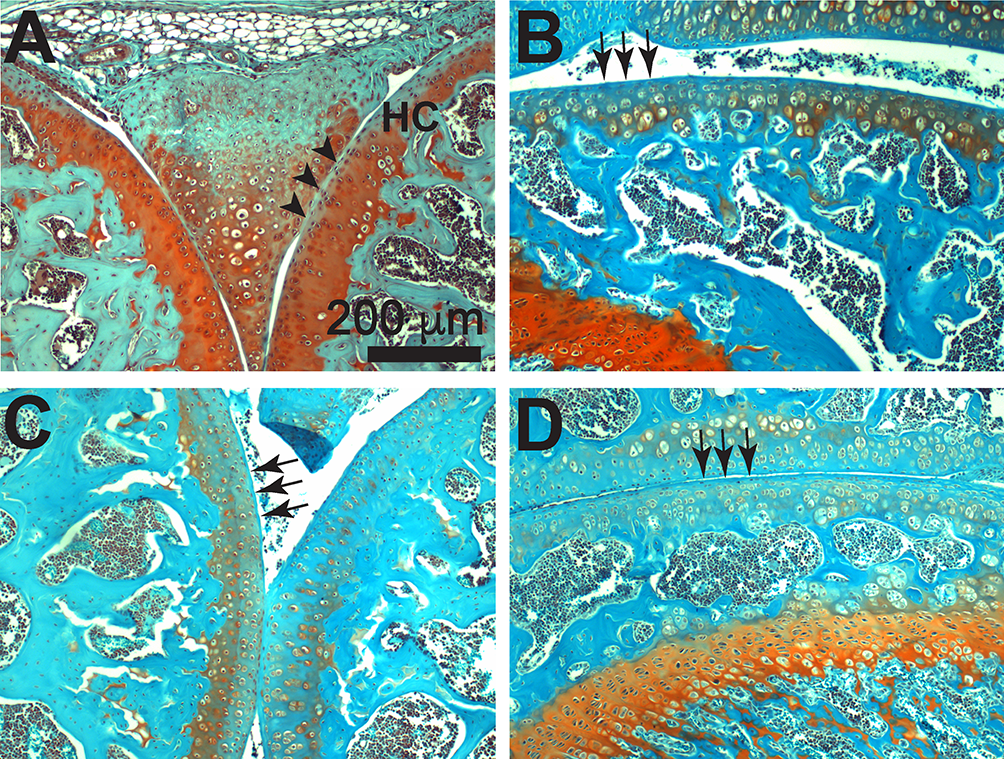

Supplement: Additional file 1: — Effect of lentivirus-mediated heterologous expression of hSPAG11B/C and APC366 in mBSA/IL-β-induced arthritis. A-D. Representative light photomicrographs of knee joint sections stained by the method of safranin O from a control animal (A), or animals submitted to mBSA/IL-1β-induced arthritis, previously injected with vehicle (B), transduced with 2 × 10^6^ TU/joint of pWPXLD-IG (C) or phSPAG11B/C (D). The arrows indicate areas of intense extracellular matrix degradation in the hyaline cartilage. The arrowheads show areas of normal hyaline cartilage. Abbreviations: JC joint cavity. (TIF 2265 kb) [file 13075_2017_1326_MOESM1_ESM.tif]
